# In Chronic Spontaneous Urticaria, Comorbid Depression Linked to Higher Disease Activity, and Substance P Levels

**DOI:** 10.3389/fpsyt.2021.667978

**Published:** 2021-05-26

**Authors:** Bachar Memet, Eren Vurgun, Fatma Barlas, Martin Metz, Marcus Maurer, Emek Kocatürk

**Affiliations:** ^1^Department of Dermatology, Koç University School of Medicine, Istanbul, Turkey; ^2^Department of Medical Biochemistry, Okmeydani Training and Research Hospital, Istanbul, Turkey; ^3^Department of Psychiatry, Okmeydani Training and Research Hospital, Istanbul, Turkey; ^4^Dermatological Allergology, Allergie-Centrum-Charité, Department of Dermatology and Allergy, Charité—Universitätsmedizin Berlin, Berlin, Germany

**Keywords:** urticaria, depression, substance-P, pathogenesis, psychiatric disorders

## Abstract

**Background:** Patients with chronic spontaneous urticaria often exhibit psychiatric comorbidities including depression that contribute to the impairment of their quality of life. How CSU and depression are linked isn't well-understood. Substance P has been shown to be increased in patients with CSU and is held to contribute to the pathogenesis of depression.

**Methods:** We measured disease activity in 30 CSU patients without depression and 30 CSU patients with depression by using the urticaria activity score. The severity of depression was assessed with the Beck Depression Inventory. We measured SP levels in these patients as well as in 30 healthy control subjects. In patients with comorbid depression, we correlated SP levels with CSU disease activity and the severity of depression.

**Results:** In CSU patients, disease activity and the severity of depression were positively linked. UAS7 values were higher in CSU patients with comorbid depression as compared to those without (*p* < 0.05). SP levels were higher in CSU patients with depression than in those without (*p* < 0.001), but was similar in all CSU patients compared to healthy controls. SP levels weren't correlated with UAS7 values in CSU patients with depression, whereas they were weakly but significantly correlated with BDI scores (*p* < 0.05).

**Conclusion:** Our results suggest that, in CSU patients with comorbid depression, CSU disease activity affects the severity of depression. CSU patients with high disease activity should be explored for comorbid depression.

## Introduction

Chronic spontaneous urticaria (CSU) is an inflammatory disease characterized by recurrent itchy wheals, angioedema or both, that appear and disappear spontaneously, usually within 24 h (1). CSU, in most patients, persists for several years before spontaneous remission (2). The etiology of CSU is, as of yet, not completely understood. It is generally held that CSU is a systemic inflammatory condition that primarily affects the skin and that autoimmune mechanisms are responsible in most of the patients. This can be either autoimmunity type I (“autoallergy”), where IgE autoantibodies and respective autoantigens degranulate skin mast cells, or autoimmunity type IIb, where mast cell-activating IgG or IgM autoantibodies directed against IgE or the IgE receptor drive the pathogenesis (3–5). In addition to these mast cell degranulating factors, modulators of mast cell activation are thought to contribute to the development of signs and symptoms in patients with CSU. Infection-associated signals, food components and also neuropeptides are seen as such relevant mast cell modulators (6). Degranulating mast cells release histamine and other inflammatory mediators from preformed granules, which leads to the activation of skin nerves (itch), vasodilatation (erythema), and increased vascular permeability (edema) and the development of wheals and angioedema (7).

A recent meta-analysis of published data concluded that almost one out of three patients with CSU has at least one concomittant psychiatric disorder (8). Among these comorbidities, depression, anxiety, and somatoform disorders are the most prevalent ones (9–14). Overall, the presence of depression in CSU patients is related to higher quality of life impairment (10, 15). Recent studies suggest that CSU drives the development of comorbid depression (16), as the rate of comorbid depression increases with the duration of CSU. Furthermore, stress, which is a major driver for developing depression, is held to contribute to the development and the severity and/or the duration of chronic urticaria (9, 17). On the other hand, long-standing symptoms of CSU can also become a source of stress, and thus for psychiatric comorbidities. Therefore, mental health evaluations and management are important elements in CSU management (18). Whether or not the symptom burden in CSU patients is linked to the severity of comorbid depression is, however, largely unknown. Most importantly, there is currently no information how CSU and depression are linked on a molecular level.

One possible candidate for linking CSU and depression is Substance P (SP). SP is expressed in various brain regions such as amygdale, dorsal raphe nucleus and frontal cortex, which are involved in response to emotional stimuli (19). SP has been suggested to play a role in the etiology of major depressive disorder, and SP antagonists have been previously studied for their potential antidepressant effects (20, 21). On the other hand, SP is known to be a powerful vasodilator causing plasma leakage and increased vascular permeability, and it can stimulate histamine secretion from mast cells (22, 23). The intradermal injection of SP results in an immediate wheal, flare and itch response as well as to an infiltration of granulocytes into the skin (24, 25). These observations and the knowledge of sensory nerve activation in urticaria are suggestive of a possible role of SP in the pathogenesis of CSU (26). In support of this, it has been reported that serum levels of SP are elevated in patients with CSU (27, 28). These findings led us to speculate that SP may be involved in linking CSU and disease activity to depression and the severity of depression, respectively, in patients with CSU.

Here, we investigated whether CSU disease activity and the severity of comorbid depression are correlated, and we explored the role of SP in this association. To this end, we measured disease activity in CSU patients with depression and assessed the severity of their depression. To better understand the role of SP, we measured serum levels of SP in 30 CSU patients each with and without depression as well as in healthy control subjects and correlated serum SP levels in CSU patients with comorbid depression with activity of CSU and severity of depression.

## Materials and Methods

### Study Conduct

This study was conducted in accordance with the Declaration of Helsinki, and ethical approval was obtained from Okmeydani Training and Research Hospital Ethics Committee of Clinical Investigations on 07.07.2015. All patients provided informed consent.

### Patient Selection and Inclusion

Between September 2015 and March 2016, CSU patients from the Department of Dermatology of Okmeydani Training and Research Hospital were assessed by use of the Beck Depression Inventory (BDI) for comorbid depression. CSU patients who scored ≥17 points in the BDI were referred to a psychiatrist. A diagnosis of depression by the psychiatrist was required for the patients to be included in the “urticaria with depression” group (*n* = 30). CSU patients who scored <17 points in the BDI were included in the “urticaria without depression” group of the study (*n* = 30). Inclusion criteria were the diagnosis of continuous CSU (longer than 6 weeks, no period without wheals and angioedema for longer than a week during the last 6 weeks) by an expert for urticaria, an age between 18 and 65 years, and the ability to document CSU signs and symptoms on a daily basis. The main exclusion criteria were severe systemic disease, continuous use of systemic corticosteroid or other immunosuppressive drugs, regular use of NSAIDs, and the use of antidepressants during the last 6 months. Patients using alternative therapies or diets that might affect the course of urticaria during or 6 weeks before the study and pregnant/breastfeeding women were also excluded.

The patients were started on antihistamine treatment (cetirizine 10 mg/days) after Autologous Serum Skin Test (ASST) and were taken to follow up. Patients were asked to complete the urticaria activity score (UAS7) before treatment and during follow-up.

A control group of 30 healthy volunteers was recruited from our Cosmetology Policlinic between December 2015 and March 2016. All healthy subjects had fewer than 17 points in the BDI and declared that they did not have any diseases according to the inclusion and exclusion criteria.

### Measurement of Substance P

In addition to routine examinations, 5 ml of blood was collected into serum separator tubes from all participants at the beginning of the study, and from the patient groups after 3 months of treatment. Immediately after blood collection, aprotinin (11 μL, 30 TIU) was added to each tube for a final concentration of 0.014 TIU/mL, and the tubes were mixed by inverting 10 times according to the kit protocol. The blood, which was allowed to stand for 30 min to coagulate, was centrifuged at 1,000 × g for 15 min. After all sera were collected, SP levels were determined by ELISA (Enzyme Linked ImmunoSorbent Assay) using the Parameter™ Substance P kit (R&D Systems Inc., Minneapolis, USA).

### Statistical Analysis

All statistical analyses were performed using SPSS 17.0 (SPSS Inc., Chicago, USA) and *p* < 0.05 was accepted as significance level. Age, duration of urticaria, UAS7 and BDI scores were not normally distributed, so they were expressed as median (25–75 percentile). Non-parametric Mann-Whitney *U* test was used to compare age, urticaria duration, UAS7, and BDI scores. Chi-square test was used when female/male ratios were compared, and Fisher's Exact test was used if necessary, conditions were not met. SP levels were not normally distributed; after logarithmic transformation was applied, the distribution was in accordance with normality (Kolmogorov-Smirnov test). As a result, SP levels were compared using logarithmically transformed data, but were expressed as true mean ± standard deviation for ease of understanding. Comparisons were made by one-way ANOVA test because the logarithmic values were normally distributed, and the variances were homogeneous (Levene's test). In *post hoc* evaluations, Tukey HSD was used when the group numbers were equal, and Bonferroni test was used when the group numbers were not equal. Correlations were examined with Spearman's test.

## Results

### In CSU Patients, Disease Activity and the Severity of Comorbid Depression Are Linked

**Figure 1:**
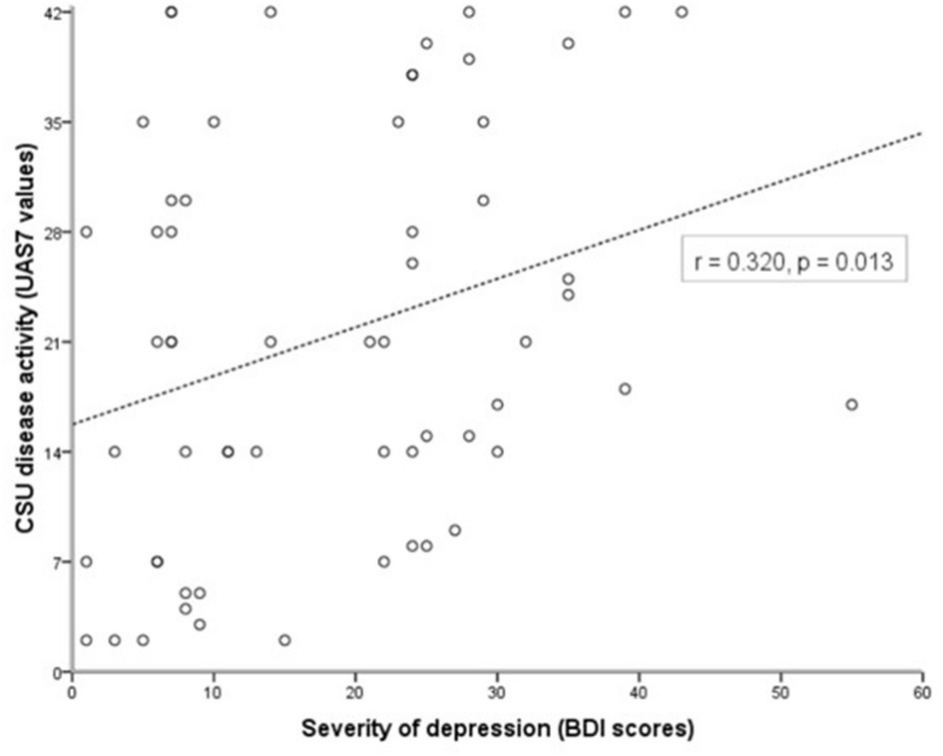
Urticaria activity and severity of comorbid depression are linked in patients with CSU. Disease activity in CSU was assessed using the weekly urticaria activity score (UAS7, 0–42), and the severity of comorbid depression was determined using the Beck Depression Inventory (BDI, 0–63; CSU patients with BDI scores ≥17 were considered as CSU with comorbid depression after confirmation by a psychiatrist). Correlation analysis shows a link between higher UAS7 and higher BDI scores (r = 0.320, *p* = 0.013).

In CSU patients with comorbid depression, urticaria disease activity and depression severity were correlated (r = 0.320, *p* = 0.013), i.e., the higher the weekly urticaria activity score (UAS7), the more severe the depression (BDI values; Figure 1). Also, urticaria activity as assessed by the UAS7 was found to be higher in CSU patients with comorbid depression as compared to those without (*p* = 0.032, Table 1). All other parameters, i.e., age, gender, duration of urticaria and ASST positivity were not significantly different between urticaria patients with and without depression. Demographic characteristics of controls and patient groups are shown in Table 1.

**Table 1:** Demographic characteristics of the patients and controls.

	**Urticaria**	**Urticaria with depression**	**Control**
*N*	30	30	30
Age	41.5 (31.7–58.2)^*^	40.5 (32.7–53.7)^**^	32 (26.7–35.7)
Female, *n* (%)	22 (73%)	28 (93%)	22 (73%)
Duration of urticaria (months)	21 (6–60)	18 (6–69)	—
ASST positive patients, *n* (%)	16 (53%)	17 (57%)	—
UAS7 values	14 (5–29)	23 (15–38)^#^	—
BDI values	7 (2.7–9.2)	27.5 (24–32.7)^**^^##^	7 (2.7–9.2)

*ASST, Autologous serum skin test; UAS7, weekly urticaria activity score; BDI, BECK depression score*.

*Age, duration of urticaria, UAS7, and BDI results as median (25–75.percentile); SP results are given as mean ± standard deviation*.

* *p < 0.05 and*

** *p < 0.001 when compared with control group*.

# *p < 0.05 and*

## *p < 0.001 when compared with urticaria group*.

### Serum Levels of Substance P Are Higher in CSU Patients With Depression Than in Those Without

**Figure 2:**
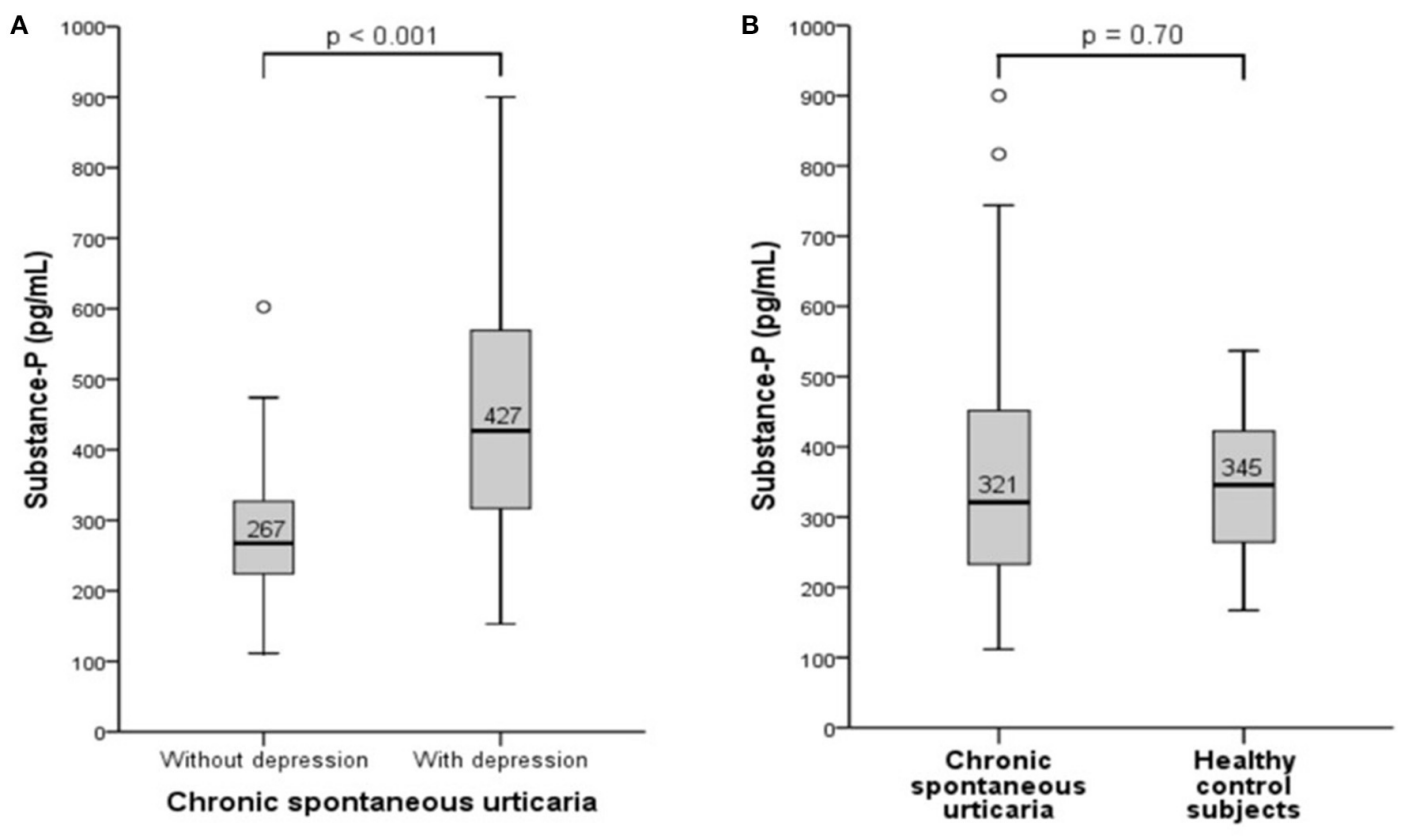
Serum levels of Substance P are higher in CSU patients with depression than in those without. Substance P was measured by ELISA and serum concentration was compared between CSU patients with and without depression **(A)** and between CSU patients and healthy controls **(B)**.

To assess whether substance P (SP) is a possible link between higher CSU disease activity and the severity of comorbid depression, we first compared serum levels of SP in CSU patients with and without depression. CSU patients with comorbid depression had 1.6-fold higher SP levels than CSU patients without depression (*p* < 0.001, Figure 2A). However, SP levels in all urticaria patients combined, i.e., CSU patients with and without depression, did not differ significantly from those in healthy controls (*p* = 0.7, Figure 2B).

### Levels of Substance P, in CSU Patients With Depression, Correlate With the Severity of Their Depression, but Not Their Urticaria Activity

**Figure 3:**
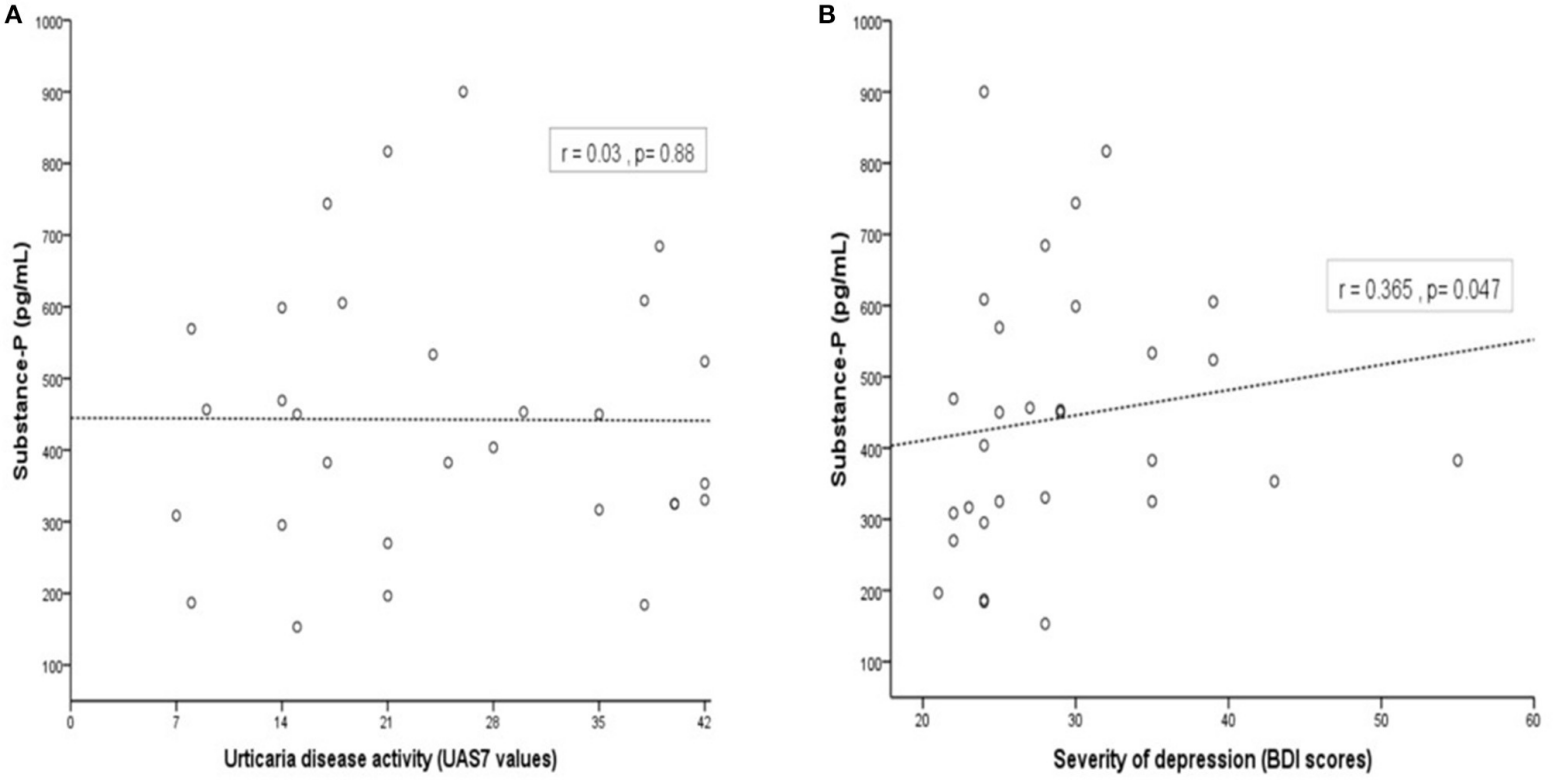
In patients with CSU and depression, SP is correlated with the severity of comorbid depression but not with CSU activity. Concentration of SP serum levels was not correlated with CSU disease activity (measured by UAS7, r = 0.03, *p* = 0.88; **(A)** and but correlated with severity of comorbid depression (assessed by BDI, r = 0.365, *p* = 0.047; **(B)**.

To further investigate the association between SP and CSU activity and the severity of the comorbid depression in CSU patients with depression, we next evaluated if SP levels are linked to CSU activity as assessed by UAS7 and to depression severity, measured by BDI. While SP levels showed no correlation with urticaria disease activity as assessed by the UAS7 (r = 0.03, *p* = 0.88; Figure 3A), SP levels were positively and significantly correlated with the severity of depression (r = 0.365, *p* = 0.047; Figure 3B).

## Discussion

In the present study, we show that in CSU patients with comorbid depression, CSU disease activity is correlated with the severity of depression and that SP levels in the serum of CSU patients with depression are linked to the severity of the comorbid depression.

The sensory neuropeptide substance P (SP) is one key mediator of an acute local stress response through neurogenic inflammation (29). Although it was identified as a peptide of neuronal origin, studies have shown that SP is also produced by inflammatory cells such as macrophages, eosinophils, lymphocytes, mast cells, and dendritic cells (30–33). SP increases lymphocyte proliferation and immunoglobulin production and stimulates the release of proinflammatory cytokines from lymphocytes, monocytes, macrophages and mast cells (34). It also increases the release of histamine and serotonin from mast cells, suggesting that SP might be involved in the pathogenesis of both urticaria and depression.

Skin mast cells are located close to sensory nerve endings and are known to be activated by neuropeptides such as SP, neurotensin (NT) and pituitary adenylate cyclase activating polypeptide (PACAP) released from human dermal neurons (35). Many dermatoses, such as atopic dermatitis and psoriasis, are reportedly triggered or exacerbated by stress (36). Pavlovic et al. (37) showed that exposure to pronounced stress and atopic dermatitis-like allergic dermatitis equipotently raise the number of cutaneous nerve fibers containing the prototypic stress neuropeptide substance P in mice. It has also been shown that neuropeptides released from sensory skin nerves in mice contribute considerably to a mast cell-mediated inflammatory skin reaction (38). These observations led to the conclusion that stress-induced local neurogenic inflammation plays a role in the pathogenesis of mast cell-driven diseases (39).

Psychological stress in CSU might act in two ways. (1) The signs and symptoms of CSU and the associated impact on the quality of life can cause an increased psychological stress, and (2) chronic stress might contribute to the development, maintenance or exacerbation of CSU. Stressful life events have been found to be higher in prevalence in patients with CU 6 months before symptom onset compared to controls and a high percentage (81%) of CU patients believe that their illness is due to stress (13, 40, 41).

In the present study, we found that UAS7 scores of patients with urticaria and depression were higher than those of patients with urticaria alone and that BDI scores are positively correlated with UAS7 scores, i.e., that the severity of comorbid depression and the urticaria disease activity are linked in patients with CSU. This observation is compatible with the observations of Tat et al. (42) who reported a significant positive correlation between UAS7 and depression scores, but differs from other studies and also a recent meta-analysis that reports no association between the presence of psychiatric illness and severity of chronic urticaria (8). Our findings indicate that either the psychiatric comorbidity seems to worsen urticaria symptoms or, vice versa, the worse the urticaria, the more the patient is depressed. This finding is important as it emphasizes the need for mental health evaluation in CSU patients and the potential benefit of interventions targeting the concurrent psychiatric disease on the improvement of urticaria symptoms.

Since we hypothesized that SP might play a role in the pathogenesis of pschychiatric disease accompanying CSU, we compared SP levels in CSU patients with and without depression. We found that serum levels of SP in CSU patients with depression were higher than those in CSU patients without depression. There are conflicting findings in the literature concerning SP levels in patients with depression. Bondy et al. (43) found that serum SP levels of major depression patients were higher than the control group, but depression patients did not show a significant change in SP levels after 4 weeks of treatment. Carpenter et al. (44) reported that SP levels in cerebrospinal fluid of treatment-resistant depression patients were lower than those of healthy controls, and SP levels did not correlate with the Hamilton Depression Rating Scale (HAMD-24). Similar to Bondy et al. (43), we found increased levels of SP in CSU patients with depression, and in contrast to Carpenter et al. (44) SP levels were positively correlated with BDI scores.

Overall, SP levels in this study were not found to be higher in CSU patients as compared to controls. Only very few previous studies investigated the role of SP in CSU. Consistent with our findings, Tedeschi et al. (45) did not find a difference between the SP levels of the urticaria and the healthy control group. This differs from the findings of Zheng et al. (46), Metz et al. (27) and Başak et al. (28) who found elevated levels of SP in CSU patients. In our study, and in contrast to the aforementioned, we included balanced populations of urticaria patients with or without depression with special focus on the impact of depression on urticaria and SP. The other studies did not select for the presence or absence of associated depression and, therefore, may have had an overrepresentation of patients with comorbid depression, possibly resulting in higher SP levels. Perhaps these two conditions, CSU and depression together, may raise serum levels of SP due to increased inflammation. Since SP levels were not found to be correlated with UAS7 scores in the present study, the increased levels of SP in urticaria patients with depression were not due to higher urticaria disease activity in these patients. On the other hand, we found that patients with depression have higher urticaria disease activity, which might be caused by increased inflammation in these patients or vice versa. Substance P has been shown to play a role in inducing inflammation in response to a variety of irritants and may act as part of central nervous system pathways involved in psychological stress (47). It is tempting to speculate that CSU coupled with depression is a particular type of urticaria, and that distinct pathways linked to SP might be involved in the pathogenesis in these patients. Clinical trials with SP antagonists in these patients might be useful to test this hypothesis (48).

In the present study among urticaria patients with depression, there were some patients with very high SP values, i.e., higher than 700 pg/ml. Anxiety, stress, or pain may be responsible for these much higher than normal levels. In this regard, it is a limitation that the scales/scores indicating the anxiety/stress/pain levels of the patients were not available. Stress can stimulate mast cell degranulation, IgE activity, and the release of neuropeptides such as SP, CGRP, and VIP that can contribute to the pathogenesis of urticaria (49). Therefore, there is a need for studies that examine the levels of other neuropeptides than SP in urticaria patients with depression.

Serum levels of SP were not associated with urticaria activity (UAS7 values) but with severity of depression (BDI scores). The finding that SP levels of urticaria patients with depression were correlated with BDI scores and the correlation of BDI scores with the UAS7 scores emphasize the possible causal relationship between SP and CSU associated with depression. This observation is in line with the results of a recent study by Schut et al. who reported that disease activity and stress are linked in a subpopulation of CSU patients (50). Here, the authors also suggested a possible causal link between CSU and mental distress and encouraged further research to confirm whether the increased levels of stress in CSU are the reason for or the result of their high disease levels (50).

The limitations of our study include the small number of patients, which means that precise conclusions can not be drawn (e.g.; SP levels of urticaria patients with depression vs. controls; see Figure 2). Another limitation is that the stress levels of the patients were not examined. Stress can modulate the activation of mast cells and aggrevate existing urticaria (51). Investigating the role of stress in CSU exacerbations and the association with SP might also be another interesting subject for further studies.

Taken together, our results suggest that serum SP might play a role in the pathophysiology of CSU associated with depression. Urticaria associated with pschyciatric comorbidities might be a distinct type of urticaria with a different underlying pathophysiology in which neuropeptides might be importantly involved. This would warrant a different approach in those patients including the possible role of pschycological interventions and antidepressant or neuropeptide-targeting treatment.

## Data Availability Statement

The raw data supporting the conclusions of this article will be made available by the authors, without undue reservation.

## Ethics Statement

The studies involving human participants were reviewed and approved by Okmeydani Training and Research Hospital Ethics Committee of Clinical Investigations on 07.07.2015. The patients/participants provided their written informed consent to participate in this study.

## Author Contributions

BM, EV, FB, and EK made substantial contributions to conception and design, acquisition of data, or analysis and interpretation of data. BM and EK drafted the article or reviewed it critically for important intellectual content. BM, EV, MMe, MMa, and EK given final approval of the version to be published. BM, EV, FB, MMe, MMa, and EK agrees to be accountable for all aspects of the work related to its accuracy or integrity. All authors contributed to the article and approved the submitted version.

## Conflict of Interest

The authors declare that the research was conducted in the absence of any commercial or financial relationships that could be construed as a potential conflict of interest.
